# Development of new cytogenetic markers for *Thinopyrum ponticum* (Podp.) Z.-W. Liu & R.-C. Wang

**DOI:** 10.3897/CompCytogen.v13i3.36112

**Published:** 2019-08-13

**Authors:** Pavel Yu. Kroupin, Victoria M. Kuznetsova, Ekaterina A. Nikitina, Yury Ts. Martirosyan, Gennady I. Karlov, Mikhail G. Divashuk

**Affiliations:** 1 Laboratory of Applied Genomics and Crop Breeding, All-Russia Research Institute of Agricultural Biotechnology, Timiryazevskaya str. 42, Moscow 127550, Russia All-Russia Research Institute of Agricultural Biotechnology Moscow Russia; 2 Center of Molecular Biotechnology, Russian State Agrarian University-Moscow Timiryazev Agricultural Academy, Timiryazevskaya str. 49, Moscow 127550, Russia Russian State Agrarian University-Moscow Timiryazev Agricultural Academ Moscow Russia; 3 Group of Aeroponic Plant Growing Technologies, All-Russia Research Institute of Agricultural Biotechnology, Timiryazevskaya str. 42, Moscow 127550, Russia Russian State Agrarian University-Moscow Timiryazev Agricultural Acade Moscow Russia

**Keywords:** *Thinopyrum ponticum*, cytogenetic markers, repetitive DNA, fluorescence *in situ* hybridization, quantitative PCR, copy number

## Abstract

*Thinopyrum ponticum* (Podpěra, 1902) Z.-W. Liu & R.-C.Wang, 1993 is an important polyploid wild perennial Triticeae species that is widely used as a source of valuable genes for wheat but its genomic constitution has long been debated. For its chromosome identification, only a limited set of FISH probes has been used. The development of new cytogenetic markers for *Th. ponticum* chromosomes is of great importance both for cytogenetic characterization of wheat-wheatgrass hybrids and for fundamental comparative studies of phylogenetic relationships between species. Here, we report on the development of five cytogenetic markers for *Th. ponticum* based on repetitive satellite DNA of which sequences were selected from the whole genome sequence of *Aegilops tauschii* Cosson, 1849. Using real-time quantitative PCR we estimated the abundance of the found repeats: P720 and P427 had the highest abundance and P132, P332 and P170 had lower quantity in *Th. ponticum* genome. Using fluorescence *in situ* hybridization (FISH) we localized five repeats to different regions of the chromosomes of *Th. ponticum*. Using reprobing multicolor FISH we colocalized the probes between each other. The distribution of these found repeats in the Triticeae genomes and its usability as cytogenetic markers for chromosomes of *Th. ponticum* are discussed.

## Introduction

*Thinopyrum ponticum* (Podpěra, 1902) Z.-W. Liu & R.-C.Wang, 1993 (=*Agropyron elongatum* Host ex P. Beauvois, 1812, 2n =10×=70) is an allodecaploid perennial grass used as a valuable pasture crop because of its heavy fibrous root system and good regrowth (Li and Wang 2009). *Th. ponticum* has high resistance to fungal and bacterial diseases, high productivity, and it is used as a donor of valuable genes because of its high cross-breeding with wheat. Such useful features as resistance to leaf and stem rust (Sepsi et al. 2008, Mago et al. 2018, Pei et al. 2018), *Fusarium* Link, 1809 head blight (Forte et al. 2014), dominant dwarfing gene (Chen et al. 2012), resistance to pre-harvest sprouting (Kocheshkova et al. 2017), yellow pigment in the endosperm (Pozniak et al. 2006), blue aleurone layer (Liu et al. 2018a), as well as perennial growth (Tsitsin 1978, Gazza et al. 2016) were transferred from *Th. ponticum* to the genome of bread wheat at the level of introgressions, additional and substituted lines, as well as partial amphidiploids by wide hybridization. Quick and accurate identification of the chromatin of *Th. ponticum* will make the use of elite genes in wheat breeding more efficient. Today, cytological methods and molecular markers are widely used to identify and monitor alien chromosomes or chromosome segments during crossing and selection.

The polyploid nature of *Th. ponticum* is still debatable, and the question about the possible origin of its subgenomes remains open to discussion. Chen et al. (1998) proposed the genomic formula JJJJsJs, and later Li and Zhang (2002) proposed the EeEbExStSt variant. The use of labeled DNA sequences as cytogenetic markers in the fluorescence *in situ* hybridization makes it possible to identify individual chromosomes and thus to establish phylogenetic relationships between genomes, understand the origin of subgenomes in allopolyploids, as well as to identify alien chromosomes in wide hybrids (Sepsi et al. 2008, He et al. 2017, Pei et al. 2018). Cytogenetic probes such as 5S rDNA (pTa794), 45S rDNA (pTa71), pAs1 (Afa family) and pSc119.2 that have become classical for bread wheat are used to identify the chromosomes of *Th. ponticum*. They are of used in the studies of the polyploid nature of *Th. ponticum* and the comparative characteristics of its subgenomes with subgenomes of closely related species (Li and Wang 2002, Brasileiro-Vidal et al. 2003). However, the available classical probes are not enough for the effective identification of chromosomes of *Th. ponticum* and it is necessary to develop new cytogenetic markers.

Repetitive DNA is the most promising for the development of chromosomal markers (Salina and Adonina 2018). Thus, new species- and chromosome-specific molecular-cytogenetic markers of *Th. ponticum* having both dispersed and tandem localization were developed (Yao et al. 2016, Liu et al. 2018a, Liu et al. 2018b). Simultaneous localization of multiple tandem repeats gives a pattern unique for each chromosome of *Th. ponticum*, different from wheat. This makes it possible to use repeats both newly identified wheatgrass-specific and common for wheatgrass and wheat in the cytogenetic characteristics of interspecific hybrids, as well as for fundamental evolutionary-phylogenetic studies (Mo et al. 2017, Komuro et al. 2013, Zhu et al. 2017; Lang et al. 2019). The development of genome-wide sequencing technologies made it possible to develop cytogenetic markers based on bioinformatic analysis of DNA sequences.

We have developed an algorithm to develop cytogenetic markers based on satellite DNA and identified five of the most copy satellite repeats in *Aegilops tauschii* Cosson, 1849 genome, which were successfully applied to A, B and D subgenomes of wheat and R of rye (Kroupin et al. 2019). In our study, this algorithm was used to study these five repeats in the genome of *Th. ponticum*. Their copy number was estimated using qPCR and FISH localization was carried out on the chromosomes of *Th. ponticum* of each of the five repeats.

## Material and methods

### Plant material

Plants of *Th. ponticum* (accession PI636523, Germplasm Research International Network, USA) were grown under optimum temperature and soil water conditions in a greenhouse.

### Real-time quantitative PCR

Genomic DNA was extracted from plants according to the protocol in Bernatzky and Tanksley (1986). Real-time quantitative PCR (qPCR) was performed as in Yaakov et al. (2013) using LightCycler instrument (Roche Molecular Systems Inc., Pleasanton, CA, USA). The primers for each monomer were designed using Primer 3.0 v 4.1.0 (http://primer3.ut.ee) based on our previously published sequences (Kroupin et al. 2019). Each reaction was performed in a 15 µl volume consisting of 2.5 µl of reaction mix containing Eva Green (Syntol LTD, Moscow, Russia), serially diluted DNA template of *Th. ponticum* (10, 2, 0.4, and 0.08 ng), and 1.0 µl each of forward and reverse primer (10 pM/µl, Table 1). A single-copy VRN1 gene was used as a reference gene as described in Yaakov et al. (2013). The relative copy number of each repeat was calculated as described in Kroupin et al. (2019).

**Table 1.** Designed primers for tandem repeats and oligonucleotide sequence used as probes (for P332 and P132).

Repeat	Primers / oligonucleotide probe
P720	F: 5’-AGCCACGTCATCAACTTTCA-3’ R: 5’-TGTCCAGTTTGTACGCGAAG-3’
P170	F: 5’-TCCTTGGAAGAATCTAGTCGTCA-3’ R: 5’-TCGGTTTTGCGCAGTGTTAA-3’
P427	F:5’-CGCCTCGACTCGCGTTACCC-3’ R:5’-GCCGAGACGAGCACGTGACA-3’
P332	F: 5’-GCTCTTCACTCGGTAGGATTT-3’ R: 5’-TCCCGTACTCGCCTAAGT-3’ BIO-5’-CGAGTGAGAGGATTGCTCTTCACTCGGTAGATTTTT-3’
P132	F:5’-TTTTACACTAGAGTTGAACTTGCTC-3’ R:5’-TGTAAAATTATTTGAACTAGGCTAT-3’ 6-FAM-5’-TTTTACACTAGAGTTGAACTTGCTCTATAGGCTAGTAC-3’

### Fluorescence *in situ* hybridization (FISH)

A chromosome spread preparation was made according to Kroupin et al. (2011) using root tips collected from living plants. FISH was carried out following the procedure in Divashuk et al. (2016). For P720, P170, and P427 probes, the amplicons produced by PCR with the primers (Table 1) were labelled using biotin (for P720) and digoxigenin (for P170 and P427) PCR labeling mix (Roche Molecular Biochemicals). For P332 and P132, the oligonucleotide probes were synthesized with 5’ end-labelled biotin (BIO) and 6-carboxyfluorescein (6-FAM), respectively (Syntol, Ltd., Moscow) (Table 1).

The detection was performed using FITC conjugated to antidigoxigenin for P170 and P427 and Cy3 conjugated to streptavidin (Roche) for P720 and P332; the chromosomes were counterstained with DAPI. Signals in all variants were visualized using an AxioZeiss Imager V1 (Carl-Zeiss, Oberkochen, Germany) fluorescence microscope with Cy3 or FITC filter. The results were recorded with an AxioCam MRm Zeiss camera (Carl-Zeiss, Oberkochen, Germany) and contrasted using AxioVision. Reprobing of the slides was performed according to Shibata and Hizume (2015) with modifications. At least four slides were prepared for each probe and at least ten chromosome spreads per slide were examined for reproducibility of the probe signals.

## Results

**Figure 1.**
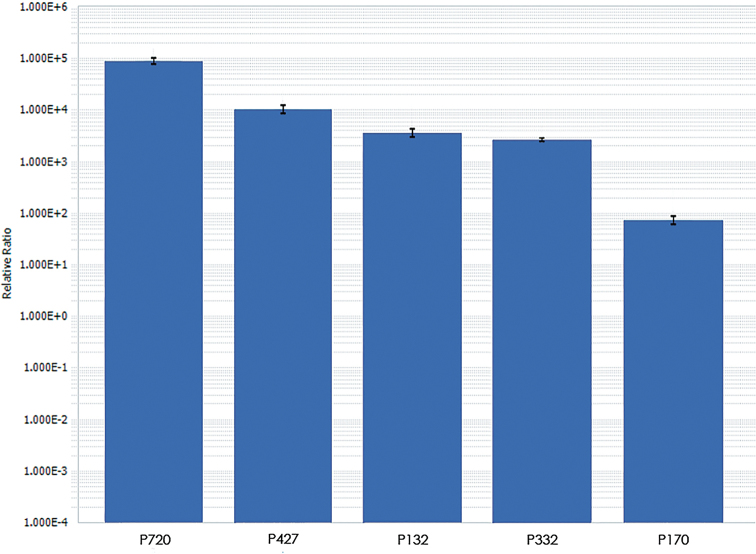
The decimal logarithm of the quantity of tandem repeats relative to the reference gene (VRN1) in *Th. ponticum* as revealed using qPCR. Error bar=standard deviation.

In order to develop cytogenetic markers for *Th. ponticum*, we chose five of the largest copy number repeats identified in the genome-wide sequence of *A. tauschii*: P720, P170, P427, P332, P132 (Kroupin et al. 2019). In order to determine the relative quantity of these repeats in the genome of *Th. ponticum*, we carried out the real-time quantitative polymerase chain reaction using primers. It was shown that repeats P720 and P427 had the largest copy number, P132 and P332 showed the average copy number, and the smallest copy number was found in P170 (Figure 1).

**Figure 2.**
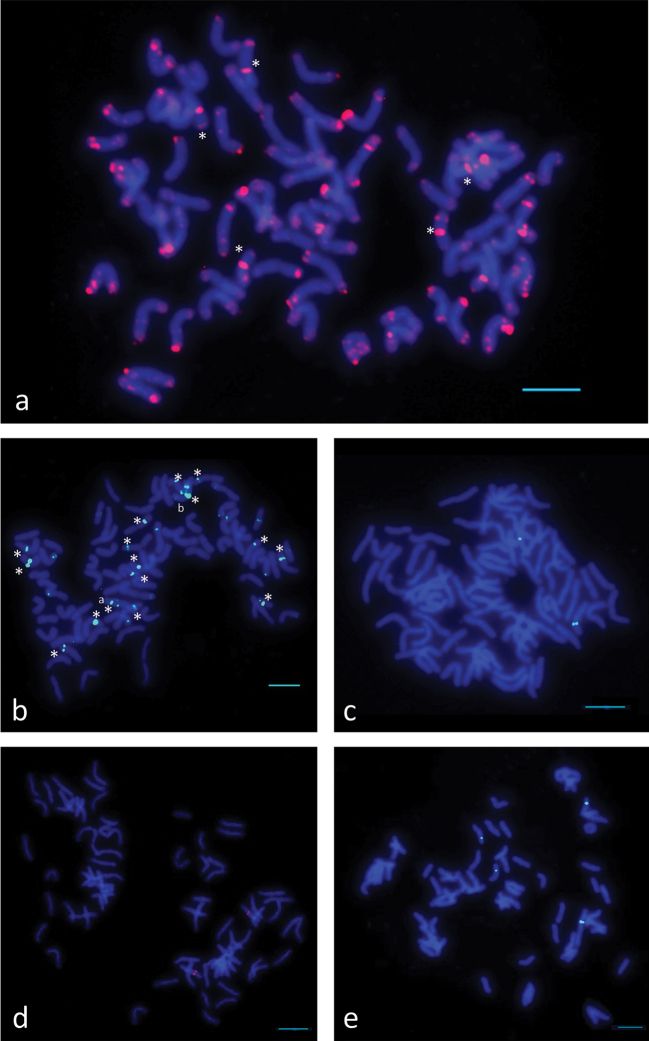
Fluorescence *in situ* hybridization in *Th. ponticum* using the following probes **a** P720 (red, asterisks show chromosomes with pericentromeric localization of P720) **b** P427 (green, asterisks show chromosomes with strong signal of P427, a and b show chromosomes with interstitial localization of P427) **c** P132 (green) **d** P332 (red) **e** P170 (green). P720, P427, and P132 are PCR products labeled with biotin (P720) and digoxigenin (P427 and P132), P332 and P170 are oligonucleotide probes labeled with biotin (P332) and 6-carboxyfluorescein (P170). Scale bar: 10µm.

As a result of the FISH procedure, five studied repeats were localized to the chromosomes of *Th. ponticum* (Figure 2).

P720 was localized to all chromosomes and the signal was of varying intensity (Figure 2a, Suppl. material 1: Figure S1). The signal was localized to terminal (telomeric and subtelomeric) regions of all chromosomes at one or both arms. Additionally, five chromosomes demonstrated the pericentromeric localization of the P720 signal (indicated by asterisks in Figure 2a and Suppl. material 1: Figure S1a).

P427 produced strong reproducible signals at 16 chromosomes that were localized partially to the pericentromeric regions and partially to the terminal regions (indicated by asterisks in Figure 2b and Suppl. material 1: Figure S2), including one chromosome with the pericentromeric and an interstitial signal at the long arm and the other chromosome with the pericentromeric and interstitial signals at both arms (chromosome a and chromosome b, respectively, Figure 2b and Suppl. material 1: Figure S2). Additionally, we detected minor faint signals at up to 24 chromosomes; however, their number varied between chromosome spreads and slides.

**Figure 3.**
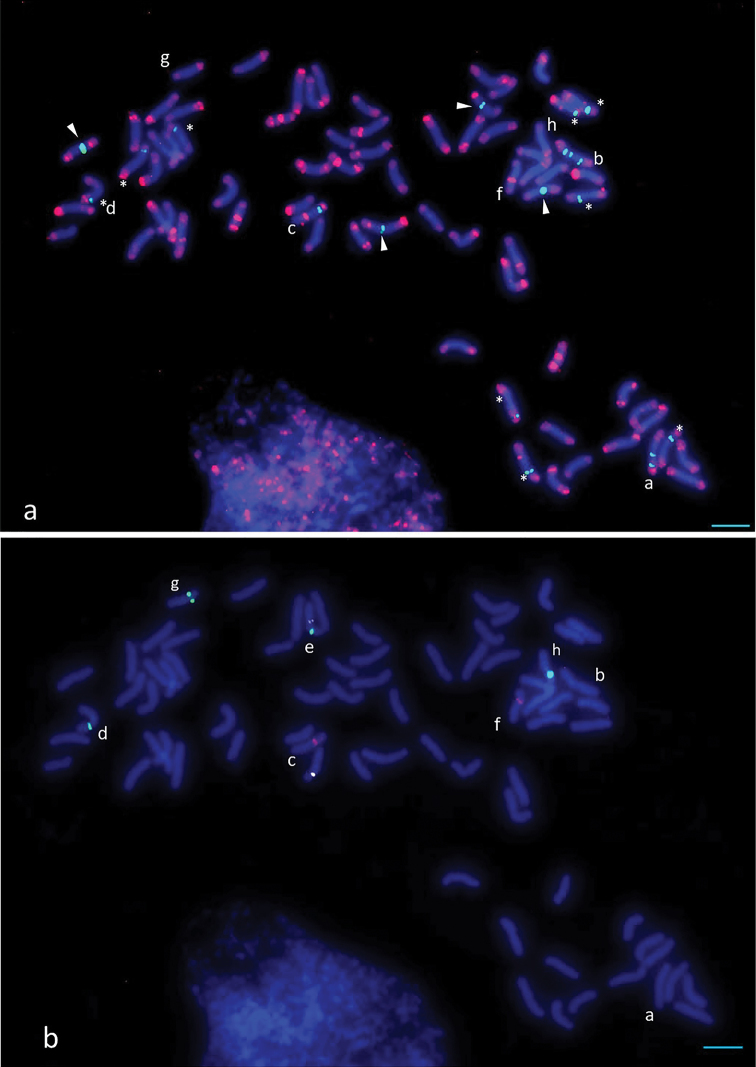
Fluorescence *in situ* hybridization in *Th. ponticum* using the reprobing multicolor FISH technique **a** P720 (red), P427 (green) **b** P332 (red), P132 (white, pseudocolor), P170 (green). P720, P427, and P132 are PCR products labeled with biotin (P720) and digoxigenin (P427 and P132), P332 and P170 are oligonucleotide probes labeled with biotin (P332) and 6-carboxyfluorescein (P170). The designation of the asterisks, arrowheads and letters (a-f) is given in the text. Scale bar: 10µm.

P132 was localized to two chromosomes in the pericentromeric region (Figure 2c, Suppl. material 1: Figure S3). P332 showed pericentromeric localization at two chromosomes (Figure 2d, Suppl. material 1: Figure S4). P170 provided a clear signal on four chromosomes: at two chromosomes in the pericentromeric region and at two chromosomes in the interstitial region of the short arm (Figure 2e, Suppl. material 1: Figure S5). To find out whether the studied repeats are colocalized to the same chromosomes of *Th. ponticum* we performed reprobing multicolor FISH at the same chromosome spreads (Figure 3, for the individual probe localization at this chromosome spread see Suppl. material 1: Figures S1–S5).

As a result of reprobing, we revealed the following groups of chromosomes: 9 chromosomes with both subtelomeric P427 and P720 signals at the same arm (Figure 3a, indicated by asterisks); 4 chromosomes with the subtelomeric P720 signal and pericentromeric P427 signals (Figure 3a, indicated by arrowheads); one chromosome with subtelomeric P720 signals at both arms and the pericentromeric and interstitial P427 signals at the short arm (Figure 3a, chromosome a); one chromosome with the subtelomeric P720 signals at both arms and the centromeric and interstitial P427 signals at both arms (Figure 3a, chromosome b); one chromosome with the pericentromeric and subtelomeric P720 signals at the long arm and the subtelomeric P427 signal (Figure 3a, chromosome c). P332 was localized to the chromosome with the pericentric and subtelomeric P720 and subtelomeric P427 signals (Figure 3, chromosome c), P170 was localized to the chromosome with the subtelomeric P720 and P427 signals (Figure 3, chromosome d), P132 and P170 were colocalized at the different arms of one and the same chromosome (Figure 3, chromosome e), P332 was localized to the chromosome with the pericentric and subtelomeric signals of P720 (Figure 3, chromosome f); two chromosomes carried both P170 and subtelomeric P720 signals (Figure 3, chromosomes g and h).

## Discussion

The development of molecular cytogenetic markers is a long and routine process. Previously, we developed an algorithm allowing to select the most likely candidates for the role of chromosomal markers based on the genome-wide sequence using qPCR (Kroupin et al. 2019). In our study, we demonstrated the effectiveness of our approach for the development of new cytogenetic markers for *Th. ponticum* based on the five repeats of *A. tauschii* of the largest copy number.

According to the results of qPCR, P720 and P427 had the highest abundance in the genome of *Th. ponticum*, which we localized to all or most of the chromosomes as a result of FISH. P720 showed a comparable copy number in bread and durum wheat and rye, which may indicate its prevalence among Triticeae (Kroupin et al. 2019). Repeats with lower copy numbers P170, P332 and P132 were localized only to a few chromosomes of *Th. ponticum*.

P720 and P427 were localized both to the terminal and pericentromeric regions of all or most of the chromosomes of *Th. ponticum*, which may indicate the conservative nature of these repeats. We also localized P720 to both the pericentromeric and terminal regions on the chromosomes of A, B and D subgenomes of wheat and R of rye (Kroupin et al. 2019). Detection of subtelomeric sequences in centromeric and pericentromeric regions is found in plants, including such grass as rice (Lee et al. 2005, Bao et al. 2006), maize (Alfenito and Birchler 1993, Jin et al. 2005) and *Agropyron cristatum* (Linnaeus, 1753) Gaertner, 1770 (Said et al. 2018). This phenomenon may be related to chromosome fusion or other ancient chromosome rearrangements (Garrido-Ramos 2015, Murat et al. 2010). At the same time, P720 and P427 showed interstitial localization on some chromosomes. The availability of subtelomeric repeating sequences in the middle of the arm is not uncommon for grasses and may be associated with heterochromatinization of interstitial regions (Komuro et al. 2013, Badaeva et al. 2016, Garrido-Ramos 2015).

P720 produced a brighter signal on the D subgenome chromosomes on the chromosomes of bread wheat (Kroupin et al. 2019). The different signal intensity of P720 and P427 in the terminal regions might also be related to the polyploid nature of *Th. ponticum* and may be important for chromosome differentiation during meiosis because telomeres play a significant role in the formation of bivalents (Garrido-Ramos 2015, Loginova and Silkova 2018). The localization of the P720 and P427 signals in both the pericentromeric and terminal sites may also be explained by the fixation of an occasional chromosomal rearrangement, since it provided differentiation of these chromosomes during meiosis. The signals of varying intensity of tandemly organized repetitive DNA were described in other closely related species, for example, in *Thinopyrum intermedium* (Host, 1805) Barkworth & D.R. Dewey, 1985 in centromere (Divashuk et al. 2016) and chromosomes of *A. cristatum* in terminal regions (Han et al. 2016).

In our work, we have shown that P170 is localized to four chromosomes of *Th. ponticum*, while the repeat itself is mostly characteristic for D subgenome of bread wheat and is absent on chromosomes of A, B and R subgenomes (Kroupin et al. 2019). Such a common hybridization may be related to the proximity of these chromosomes to the D subgenome of bread wheat. Most of the chromatin introgressions with valuable genes of *Th. ponticum* occur in the chromosome of the D subgenome of *Triticum aestivum* Linnaeus, 1753, which may be related to its proximity to the genome of *Th. ponticum* (Chen et al. 2012). Since the PCR product obtained using primers developed for the repeat monomer and genomic DNA of the studied species was used for the FISH procedure, differences in the localization and intensity of the repeat signal on the chromosomes of *Th. ponticum* in the present study and bread wheat and hexaploid triticale in Kroupin et al. (2019) may be associated with different copy numbers of the repeats on chromosomes of different species (quantitative differences), as well as with differences in the sequence of monomers (qualitative differences).

P332, P170, and P132 were hybridized on several (from two to four) chromosomes of *Th. ponticum*. P170 was hybridized specifically on chromosomes of the D subgenome, and P332 was not identified in the R subgenome of rye (Kroupin et al. 2019). Such species specificity and hybridization on a small number of chromosomes of *Th. ponticum* allows us to consider these repeats as good candidates for chromosome-specific markers. FISH reprobing procedure enabled us to distinguish individual chromosomes on the basis of the combination of the developed probes. Chromosomes a-h (Figure 3) can be univocally identified in the studied accession of *Th. ponticum* using our cytogenetic markers and the probes can be applied for the comparison of different *Th. ponticum* chromosomes among accessions of *Th. ponticum* for population studies and may be useful in studies of wide hybrids of wheat in combination with the classical FISH probes of wheat.

We colocalized the developed probes using reprobing FISH and revealed that the chromosome set of *Th. ponticum* is imbalanced: chromosomes a-f (Figure 3) have no pairs and the pattern of the signal localization is unique for each of these chromosomes. P720 was localized to the pericentromeric region at five chromosomes while P427 produced an interstitial signal at the short arm of one chromosome and at both arms of the other chromosome. This may be a peculiarity of a given accession of *Th. ponticum* and may be a result of chromosomal rearrangements at meiosis because of homeological pairing between the chromosomes. Alternatively, such uneven distribution of the signals may be the result of ancient chromosomal rearrangements that have been fixed by the natural selection. The imbalanced chromosome constitution may not be an obstacle for propagation of a given *Th. ponticum* plant since it can be propagated both by sexual and vegetative means and a given chromosome combination may be advantageous in a particular environment.

In conclusion, using our algorithm, we selected five repeats, two highly abundant repeats with localization on all or most of the chromosomes, and three repeats with a lower copy number with localization on several individual chromosomes. The repeats we selected can be used as a tool for the comparative cytogenetics of Triticeae to study phylogenetics and evolutionary relationships between species.

## Supplementary materials

Supplementary material 1 Figures S1–S5 Data type: Adobe Acrobat Document (.pdf) Explanation note: Supplementary Figure 1. Fluorescence *in situ* hybridization in *Th. ponticum* using P720 as a probe (red, PCR product labeled with biotin). Supplementary Figure 2. Fluorescence *in situ* hybridization in *Th. ponticum* using P427 as a probe (green, PCR product labeled with digoxigenin). Supplementary Figure 3. Fluorescence *in situ* hybridization in *Th. ponticum* using P132 as a probe (white pseudocolor, PCR product labeled with digoxigenin). Supplementary Figure 4. Fluorescence *in situ* hybridization in *Th. ponticum* using P332 as a probe (red, oligonucleotide labeled with biotin). https://binary.pensoft.net/file/325383This dataset is made available under the Open Database License (http://opendatacommons.org/licenses/odbl/1.0/). The Open Database License (ODbL) is a license agreement intended to allow users to freely share, modify, and use this Dataset while maintaining this same freedom for others, provided that the original source and author(s) are credited.Pavel Yu. Kroupin, Victoria M. Kuznetsova, Ekaterina A. Nikitina, Yury Ts. Martirosyan, Gennady I. Karlov, Mikhail G. Divashuk
