# Supplementary material for: Development of new cytogenetic markers for Thinopyrum ponticum (Podp.) Z.-W. Liu & R.-C. Wang
Source: Comp Cytogenet. 2019 Aug 13;13(3):231–43. doi: 10.3897/CompCytogen.v13i3.36112 (PMC6702164; doi:10.3897/CompCytogen.v13i3.36112)
Supplement: Supplementary material 1 [file comparative_cytogenetics-13-231-s001.pdf]

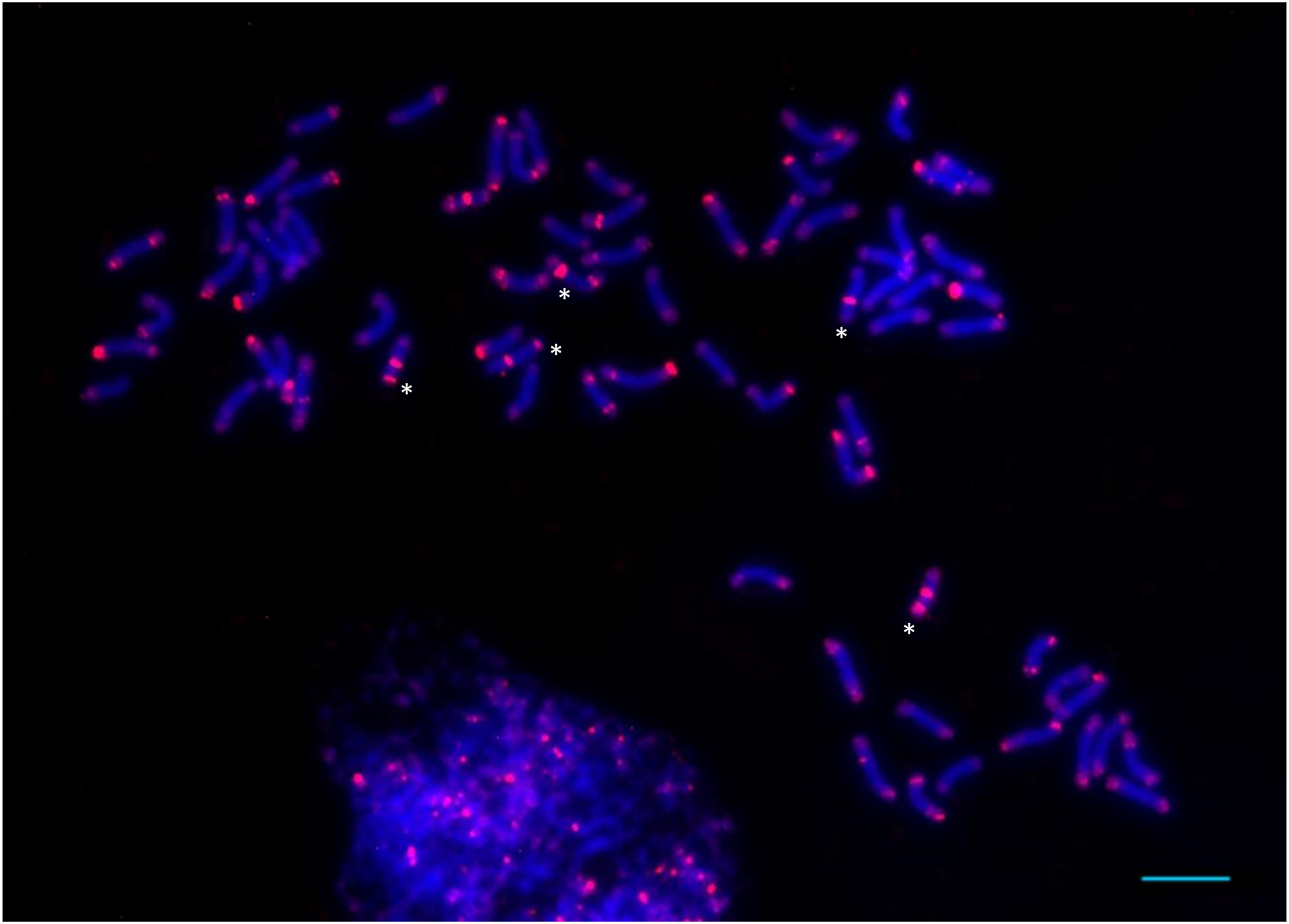

Supplementary Figure 1. Fluorescence *in situ* hybridization in *Th. ponticum* using P720 as a probe (red, PCR product labeled with biotin). Asterisks show chromosomes with pericentromeric localization of P720. Scale bar=10μm.

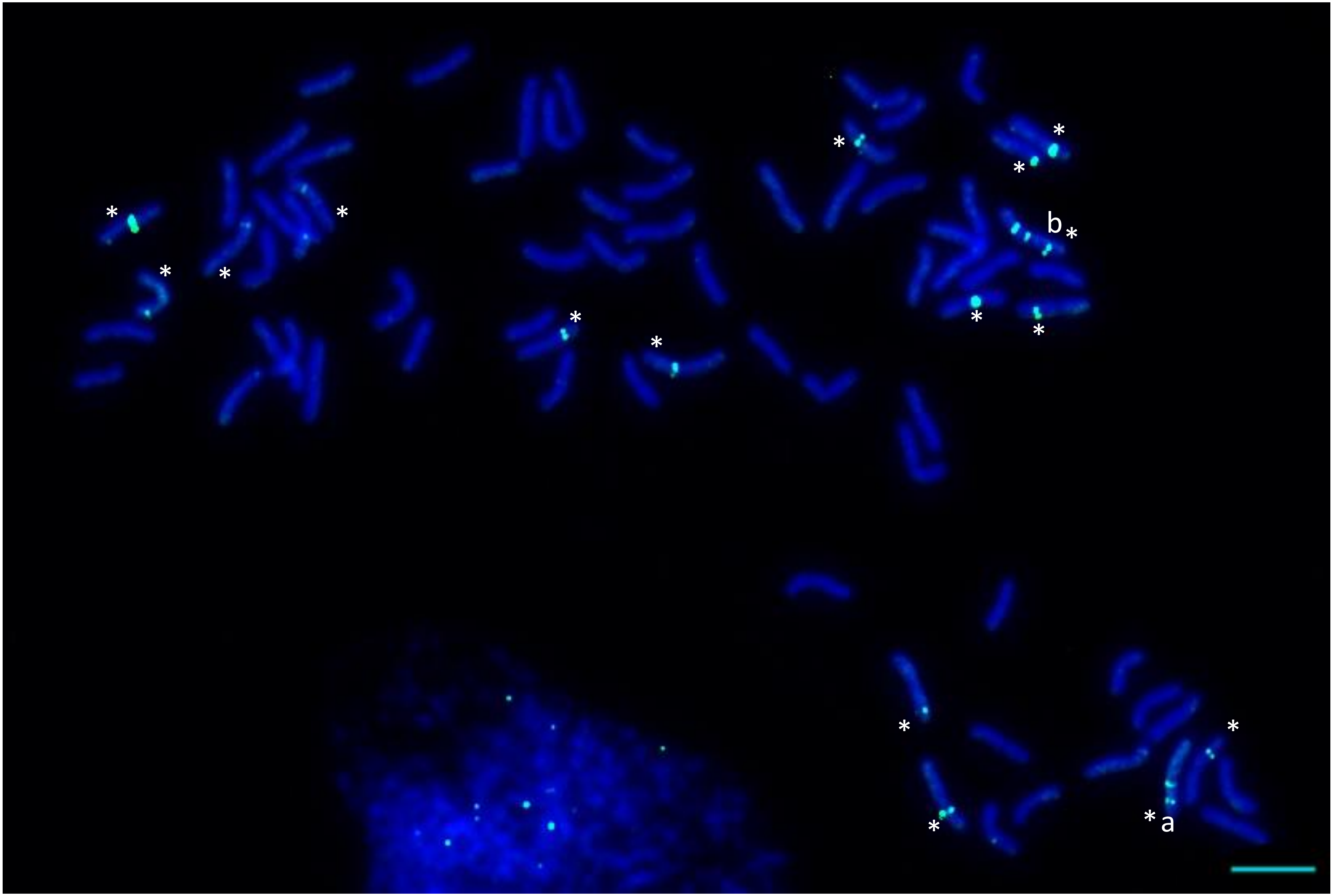

Supplementary Figure 2. Fluorescence *in situ* hybridization in *Th. ponticum* using P427 as a probe (green, PCR product labeled with digoxigenin). Asterisks show chromosomes with the strong signal of P427, a and b show chromosomes with interstitial localization of P427. Scale bar=10μm.

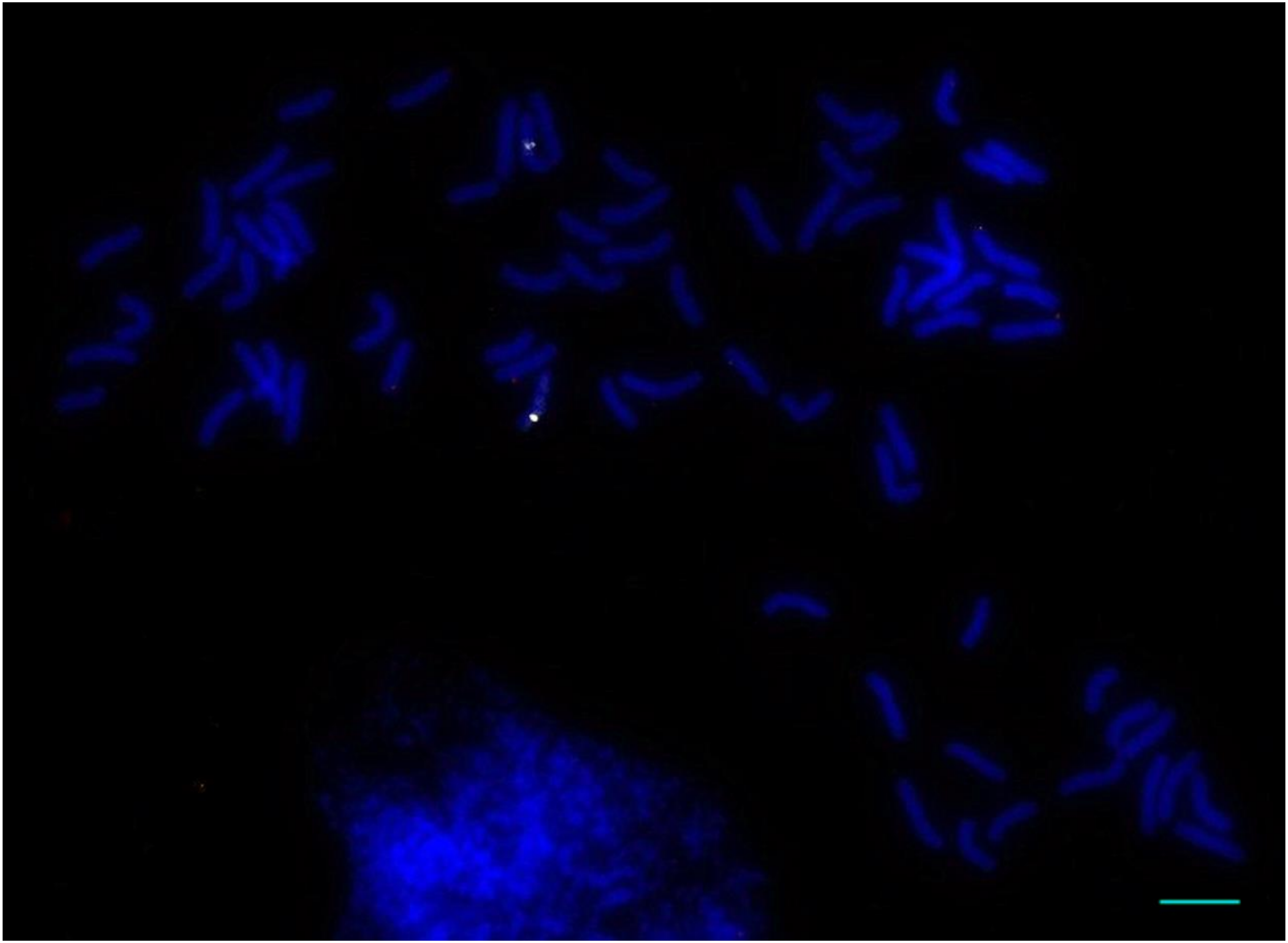

Supplementary Figure 3. Fluorescence *in situ* hybridization in *Th. ponticum* using P132 as a probe (white pseudocolor, PCR product labeled with digoxigenin). Scale bar=10μm.

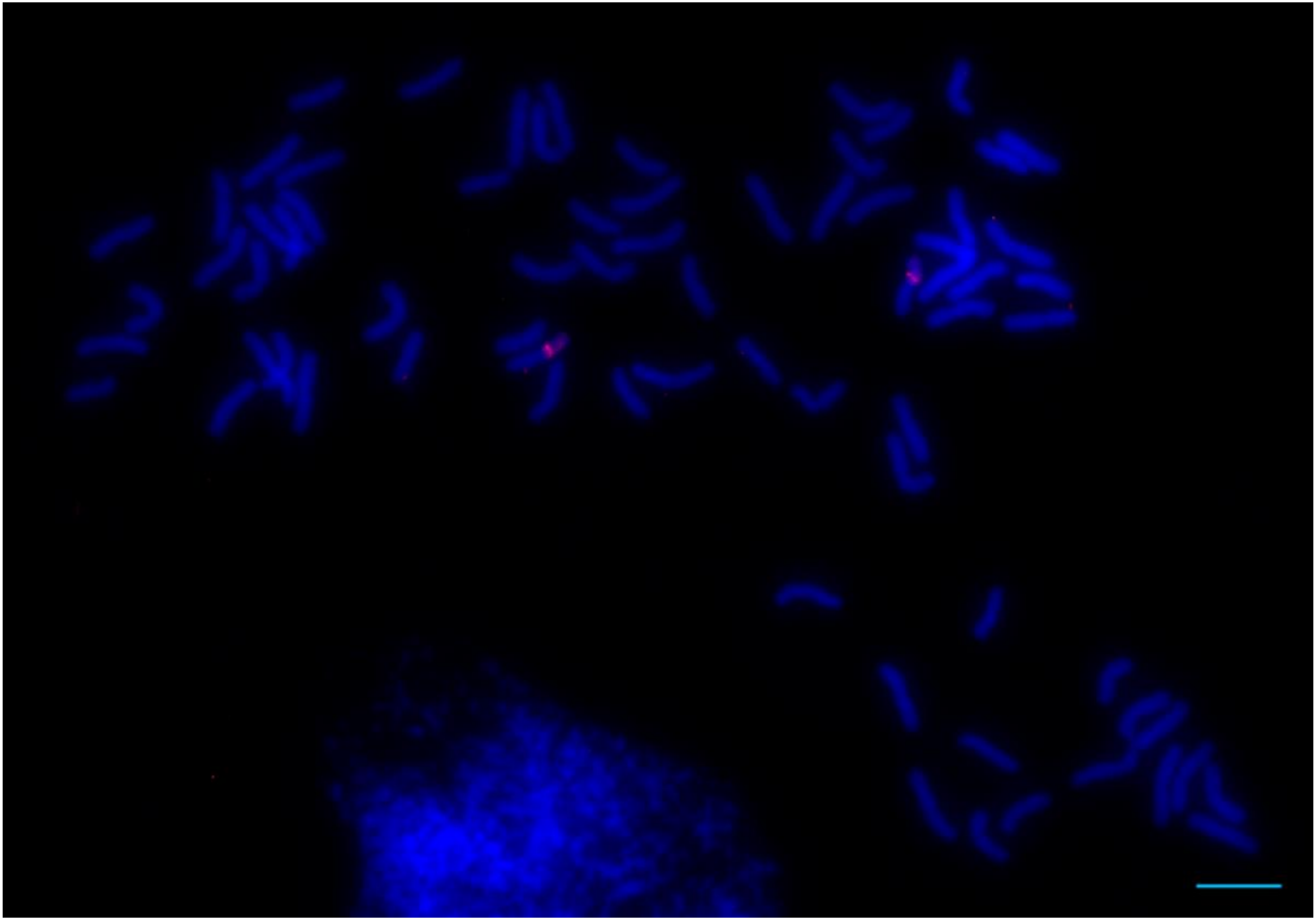

Supplementary Figure 4. Fluorescence *in situ* hybridization in *Th. ponticum* using P332 as a probe (red, oligonucleotide probe labeled with biotin). Scale bar=10μm.

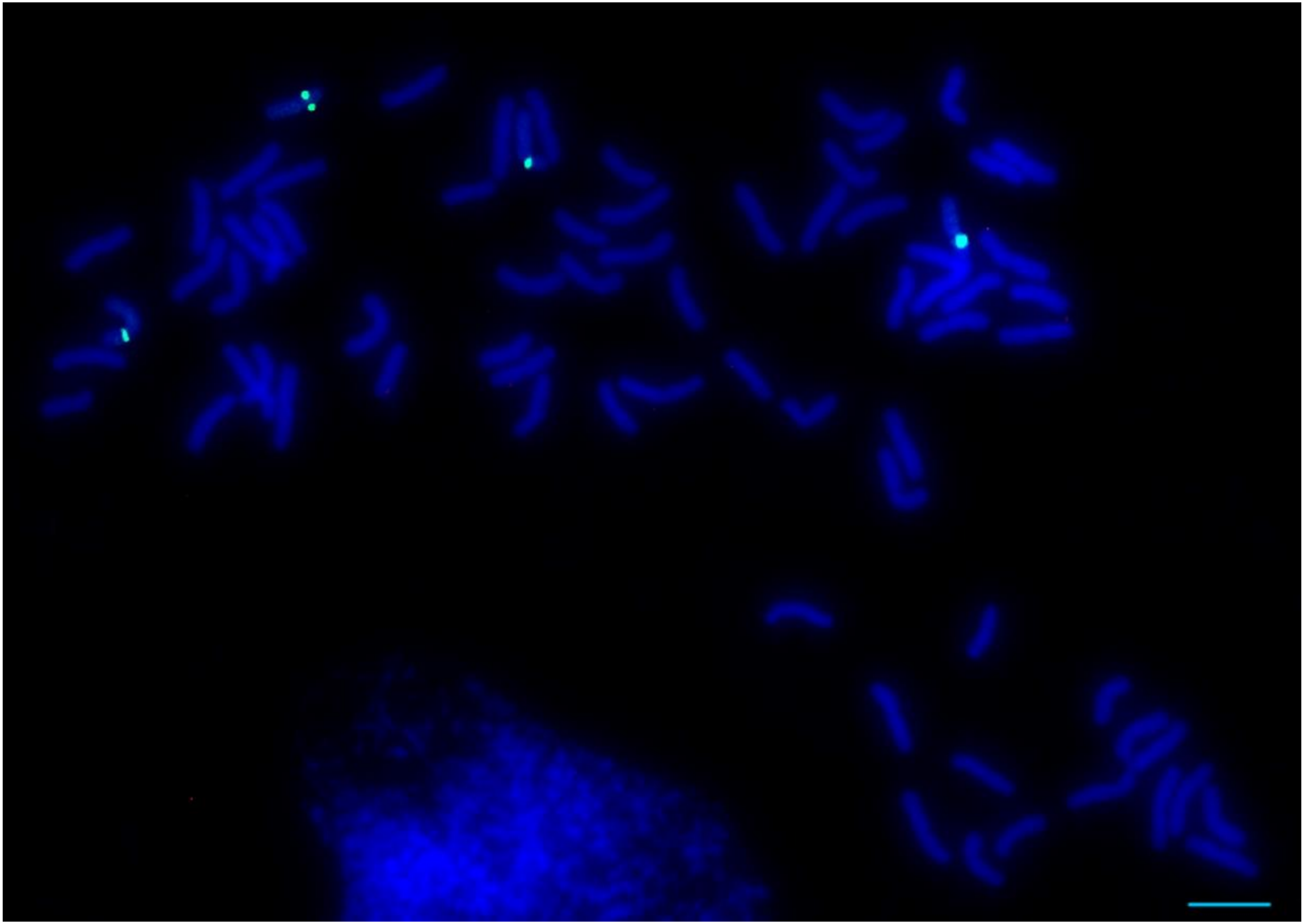

Supplementary Figure 5. Fluorescence *in situ* hybridization in *Th. ponticum* using P170 as a probe (green, oligonucleotide probe labeled with 6-carboxyfluorescein). Scale bar=10µm.
